# Prognostic Factors, Survival Analyses and the Risk of Second Primary Cancer: A Population-Based Study on Burkitt Lymphoma/Leukemia

**DOI:** 10.3390/diseases9020043

**Published:** 2021-06-15

**Authors:** Ana M. Della Rocca, Fernanda S. Tonin, Mariana M. Fachi, Alexandre F. Cobre, Vinicius L. Ferreira, Letícia P. Leonart, Giovanna Steffenello-Durigon, Joanita A. G. Del Moral, Luana Lenzi, Roberto Pontarolo

**Affiliations:** 1 Pharmaceutical Sciences Postgraduate Program, Federal University of Paraná, Curitiba 80210-170, PR, Brazil; anamdrocca@gmail.com (A.M.D.R.); fer_stumpf_tonin@hotmail.com (F.S.T.); marianamfachi@gmail.com (M.M.F.); alexandrecobre@gmail.com (A.F.C.); vinicius_lins1991@hotmail.com (V.L.F.); leticialeonart@gmail.com (L.P.L.); 2 Hematology Service, University Hospital Professor Polydoro Ernani de São Thiago, Federal University of Santa Catarina, Florianópolis 88036-800, SC, Brazil; giovanna.steff@gmail.com (G.S.-D.); joanitadm@gmail.com (J.A.G.D.M.); 3 Department of Clinical Analyses, Federal University of Paraná, Curitiba 80210-170, PR, Brazil; luanalnz@yahoo.com.br; 4 Department of Pharmacy, Federal University of Paraná, Curitiba 80210-170, PR, Brazil

**Keywords:** Burkitt lymphoma, SEER program, prognosis, survival analysis, neoplasms, second primary

## Abstract

Burkitt lymphoma/leukemia (BL/L) is an aggressive oncohematological disease. This study evaluated the population-based prognosis and survival on BL/L as well as if BL/L behaved as a risk factor for the development of second primary cancers (SPCs) and if other first tumors behaved as risk factors for the occurrence of BL/L as an SPC. A retrospective cohort using the Surveillance, Epidemiology and End Results (SEER) Program (2008–2016) was performed. Kaplan–Meier, time-dependent covariate Cox regression and Poisson regression models were conducted. Overall, 3094 patients were included (median, 45 years; IQR, 22–62). The estimated overall survival was 65.4 months (95% CI, 63.6–67.3). Significantly more deaths occurred for older patients, black race, disease at an advanced stage, patients without chemotherapy/surgery and patients who underwent radiotherapy. Hodgkin lymphomas (nodal) (RR, 7.6 (3.9–15.0; *p* < 0.001)), Kaposi sarcomas (34.0 (16.8–68.9; *p* < 0.001)), liver tumors (3.4 (1.2–9.3; *p* = 0.020)) and trachea, mediastinum and other respiratory cancers (15.8 (2.2–113.9; *p* = 0.006)) behaved as risk factors for the occurrence of BL/L as an SPC. BL/L was a risk factor for the occurrence of SPCs as acute myeloid leukemias (4.6 (2.1–10.4; *p* < 0.001)), Hodgkin lymphomas (extranodal) (74.3 (10.0–549.8; *p* < 0.001)) and Kaposi sarcomas (35.1 (12.1–101.4; *p* < 0.001)). These results may assist the development of diagnostic and clinical recommendations for BL/L.

## 1. Introduction

Burkitt lymphoma (BL) is an aggressive B-cell non-Hodgkin lymphoma (NHL) with an extremely short doubling time that often affects the extranodal sites or manifests as acute leukemia (Burkitt leukemia: BLK) [1]. The diagnosis of this malignancy is based on clinical, morphological, immunophenotypic and genetic features, yet the disease’s classification may eventually change given new research findings. Many classification systems have been proposed [2] with the *World Health Organization Classification of Tumors of Haematopoietic and Lymphoid Tissues* (WHOc) being the most currently used, with previous publications in 2001 [3], 2008 [4] and 2016 [1,5]. The BL cases classified as an atypical morphological variant in WHOc 2001 were called ‘Burkitt-like lymphoma’ [3]. Subsequently, in WHOc 2008, these cases were reallocated into the category ‘B-cell lymphoma, unclassifiable, with features intermediate between diffuse large B-cell lymphoma and Burkitt lymphoma’ (DLBCL/BL) [4] and in WHOc 2016 they were reclassified to the category ‘high-grade B-cell lymphoma’, which usually has a more aggressive clinical course and a worse prognosis than BL [1,5].

Three epidemiological clinical variants of BL exist. Endemic BL (eBL) occurs in equatorial Africa and malaria-endemic regions, being the most common childhood malignancy (5–10 cases per 100,000 inhabitants). Sporadic BL (sBL) occurs worldwide, mainly in children and young adults (2–3 cases per 1,000,000 inhabitants) and immunodeficiency-associated BL (iBL) is usually related to HIV-positive individuals (6 cases per 1000 AIDS cases) [1,6,7]. sBL patients from high-income countries (e.g., with support for intensive chemotherapy) have higher survival rates (70% to over 90%) [8,9,10,11], which is not the scenario in low-income countries, probably due to their limited therapeutic resources. These cases usually require chemotherapy protocol simplification, which may lead to lower survival rates (around 60%) [12,13].

Cross-sectional and cohort studies are important tools for identifying the distribution of a specific disease in a population to explain possible factors related to its cause and diseases outcomes and to understand the interference of social and clinical factors in the disease risk [14]. Previous epidemiological studies [15,16] on Burkitt lymphoma/leukemia (BL/L) have investigated population-based prognostic and risk factors [17,18], which include older age, black race and advanced staging. To the best of the authors’ knowledge, this is the first study in the literature evaluating patients who developed BL/L as second primary cancer (SPC) as well as other SPCs after this hematological malignancy. These data could contribute towards the early detection of other malignancies and promote tailored treatments [17,18,19]. Additionally, few registries or databases have been previously evaluated, with a small number of variables being assessed. One of the most recognized sources of information on cancer incidence and survival in the United States of America (USA) is the Surveillance, Epidemiology and End Results (SEER) Program of the National Cancer Institute, which currently covers approximately 35% of the USA population through its population-based cancer registries [20].

Thus, in addition to evaluating the prognosis and survival of BL/L patients, the main aim of this study was to evaluate the primary cancers that behaved as risk factors to the development of BL/L as an SPC as well as the risk of BL/L providing the occurrence of other SPCs in a time period from 2008 to 2016 in the USA using the SEER Program database.

## 2. Materials and Methods

### 2.1. Study Design, Data Source and Cohort Selection

This was an observational, analytical and retrospective cohort study. Data were collected from the SEER Program database (submitted data in November 2018, with information available in April 2019) [21]. The incidence (rates crude) was estimated using the SEER Program database [22], which considered the Katrina/Rita population adjustment. SEER*Stat v.8.3.5 [23] was used to collect the data and incidence calculation. Patients diagnosed with BL/L between 2008 and 2016 according to the WHOc 2008 (or later version) were included in the descriptive, prognostic factors and survival analyses (*n* = 3094). For risk factors analyses, patients with any cancer other than BL/L in this same date restriction were also included (*n* = 4,303,574). Specifically for the incidence calculation, the total population used by SEER*Stat was of 786,813,193 and the displayed rate was cases per 1,000,000.

### 2.2. Variables

The variables were extracted as follows: age at diagnosis, Ann Arbor staging, chemotherapy, histology/behavior (International Classification of Diseases for Oncology, 3rd edition: ICD-O-3), origin, primary site, primary cancer by international rules (PIR), race, radiotherapy, record number, sex, site (ICD-O-3/WHO 2008), surgery of primary site, survival (months), vital status and year of diagnosis. The definition of the variables according to the specific document from the SEER Program [24] is available in the Supplementary Materials, Data S1. Four age ranges were defined by the authors (0–19, 20–39, 40–59 and ≥ 60 years old). The variables of the primary site, surgery of the primary site and radiotherapy were regrouped according to their similarities and the number of events (Supplementary Materials, Data S2).

### 2.3. Statistical Analyses

All statistical analyses were performed using SPSS^®^ v.20 software (IBM^®^, Chicago, IL, USA). The risk relative (RR) calculation of the bivariate analysis was performed using BioEstat^®^ v.5.3 software (Pará, Brazil; Maryland, IL, USA). Values of *p* < 0.05 were considered statistically significant for all analyses and point estimates are shown along with the 95% confidence interval (CI).

#### 2.3.1. Descriptive Data

Descriptive statistical analyses were performed. The Kolmogorov–Smirnov test showed the non-normal distribution of the majority of the continuous variables, which were reported as the median, interquartile ranges (IQR—25%; 75%) and minimum and maximum values (range). The normally-distributed continuous variables were reported as the mean, standard deviation (SD) and standard error (SE). The categorical variables were reported as their absolute and relative frequency.

#### 2.3.2. Prognostic Factors and Survival Analyses

The Kaplan–Meier method was performed to estimate the survival of the patients. The outcome was the overall survival (OS) (dependent variable), represented through the ‘vital status’ variable. The mean OS survival time (in months) was estimated by the interval between the date of the BL/L diagnosis until the date of the patients’ death (due to this disease or other causes) or right censorship (patients who remained alive with or without BL/L until the end of the follow-up). The statistical comparison of the Kaplan–Meier curves at different moments of the study follow-up was assessed through Breslow (generalized Wilcoxon), Tarone–Ware and log rank (Mantel–Cox) tests.

A time-dependent covariate Cox regression model (with bivariate and multivariate analyses) was used to investigate the prognostic factors associated with the OS. This model type was selected due to the lack of proportionality observed in the survival curves (e.g., crossing) from the Kaplan–Meier graphs [25]. The size of the effects of the prognostic factors was quantified through the hazard ratio (HR). The Enter method was applied as a statistical model.

#### 2.3.3. Risk Factor Analyses for the Development of Second Primary Cancers (SPCs)

The variable ‘record number’ was chosen for the risk factor analysis. This variable sequentially numbers the tumors of a person, which has its ordered values based on the date of diagnosis and then the sequence number [24]. Only cancer numbers 1 (first cancer) and 2 (second cancer) were chosen for the ‘cases’ (BL/L) and ‘controls’ (any cancer other than BL/L) to avoid the influence of other cancers that occurred in different orders in the lifetime of a patient.

The International Agency for Research on Cancer (IARC) defines primary cancer as the one that originates in a primary site or tissue and is not an extension nor a recurrence nor a metastasis [26]. In our analysis, aiming only to evaluate primary cancers, the SEER variable called ‘primary cancer by international rules’ (PIR), which was created using IARC multiple primary rules, was used. This variable contains the category ‘excluded from IARC’, which excluded from the IARC multiple primary algorithm due to behavior. As the PIR variable did not include benign tumors or non-bladder in situ tumors in the algorithm, the ‘excluded from IARC’ category was not included in our analyses. In the SEER Research Data Record Description document, there is a note informing that for bladder tumors only, all in situ are converted to invasive tumors [24].

We evaluated which first primary cancers acted as risk factors for BL/L occurrence as an SPC (‘cases’, *n* = 148; ‘controls’, *n* = 334,597) and also if BL/L (as a first primary cancer) behaved as a risk factor for the occurrence of other SPCs (‘cases’, *n* = 66; ‘controls’, *n* = 155,234). An SPC is defined as a new primary cancer that occurs in a person who has had cancer in the past and it may occur months or years after the original (primary) cancer was diagnosed and treated [27].

A bivariate analysis was performed through an RR calculation. Cancers with a *p*-value up to 0.25 were selected as independent variables for an interrelated primary cancer risk for BL/L and were included in the Poisson regression model. For a first primary cancer, the dependent variable was the presence or absence of BL/L as an SPC. On the other hand, for the risk of any other SPCs other than BL/L, the dependent variable was the presence or absence of BL/L as a first cancer. The independent variables were tested in different combinations to obtain the final model, which was built with the type of main effects and Wald statistics.

## 3. Results

### 3.1. Population Characteristics

A total of *n* = 3094 patients were diagnosed with BL/L in the USA from 2008 to 2016 (mean 343.8 ± 29.9 new cases per year). The estimated incidence of the disease was 3.9 cases per 1,000,000 inhabitants (SE, 0.1; 95% CI, 3.8–4.1) [22]. The median survival time was 19 months (IQR, 4.25–57.0; range, 0–107). During this follow-up, 1,907 (61.6%) patients remained alive. The median age at diagnosis was 45.0 years (IQR, 22.0–62.0; range, 0–97) with two peaks of BL/L incidence: 5–9 years (*n* = 231; 7.5%) and 45–49 years (*n* = 240; 7.8%) (see Supplementary Materials, Data S3 and Figures S1 and S2). According to the ICD-O-3 and the WHOc 2008, 2685 (86.8%) patients had BL (ICD-O-3: 9687/3), of whom 1849 (59.8%) had a nodal presentation and 836 (27.0%) had an extranodal presentation. Overall, 409 (13.2%) patients were classified as having BLK (ICD-O-3: 9826/3). Other characteristics are shown in Table 1.

### 3.2. Influence of Prognostic Factors on the Overall Survival (OS)

**Figure 1:**
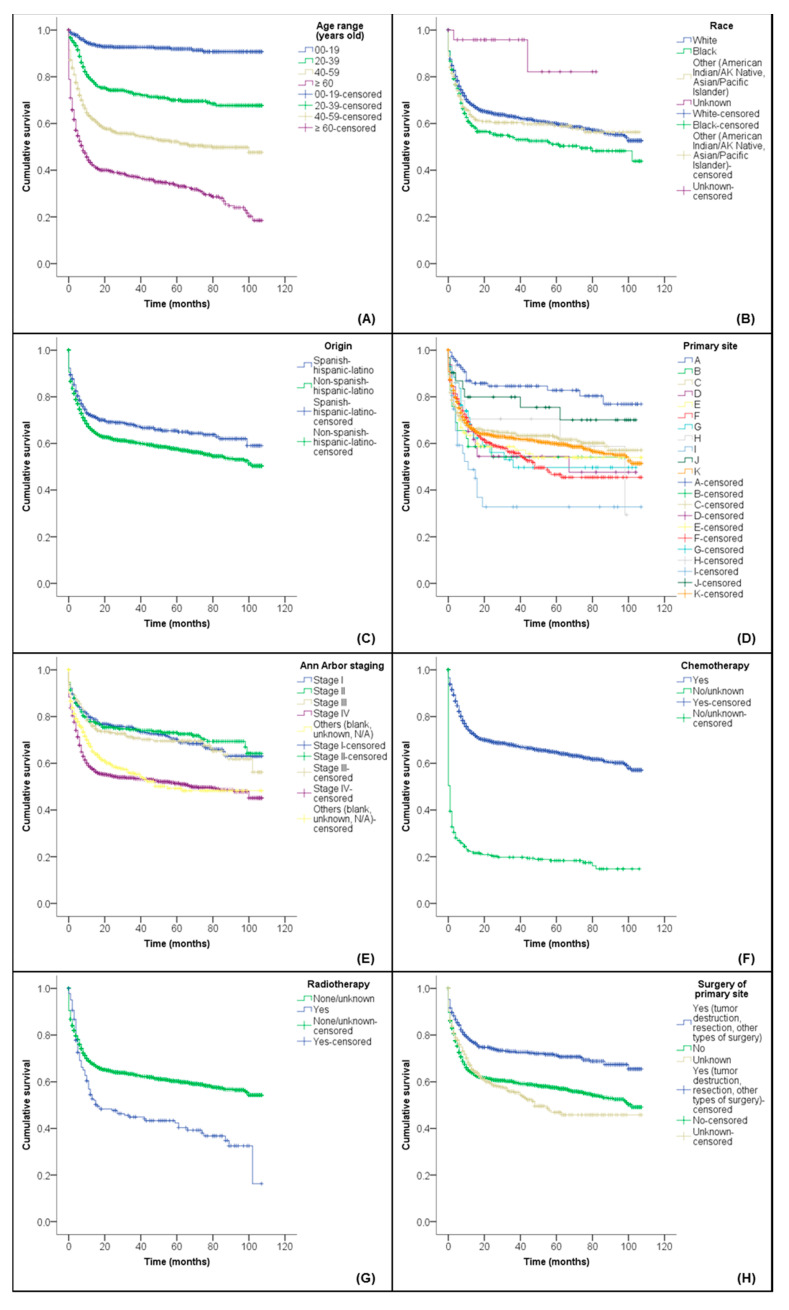
Cumulative survival probability from BL/L according to (**A**) age range, (**B**) race, (**C**) origin, (**D**) primary site, (**E**) Ann Arbor staging, (**F**) chemotherapy, (**G**) radiotherapy and (**H**) surgery of the primary site. Abbreviations: BL/L, Burkitt lymphoma/leukemia; CNS, central nervous system; GIT, gastrointestinal tract; N/A, not applicable. Notes: all variables were significant (*p*-value < 0.05) for all three statistical tests (Breslow (Generalized Wilcoxon), Tarone–Ware and log rank (Mantel–Cox)) applied in the analyses. The captions for the ‘primary site’ variable groups (Figure 1D) are as follows: A, lip, oral cavity and pharynx; B, unknown primary site and other and ill-defined sites; C, GIT and attachments; D, respiratory system and cardiac system and attachments; E, skin, bones, joints, articular cartilage and other tissues/sites; F, hematopoietic and reticuloendothelial systems; G, breast and female/male genital organs; H, urinary system; I, eye, adnexa, brain and other parts of the CNS; J, glands (thyroid and adrenal); K, lymph nodes.

A total of 10 out of 3094 patients were considered as missing by the SEER*Stat software. Thus, a survival analysis was performed with 3084 patients. In the 108 months of follow-up (2008 to 2016), a total of 1177 deaths (38.2%) for any cause occurred. The mean OS was 65.4 months (SE ± 0.9; 95% CI, 63.6–67.3). Figure 1 shows Kaplan–Meier graphs for the significant variables of the OS. See Supplementary Materials, Data S4 for the complete results of the Kaplan–Meier analysis (Table S2).

The time-dependent Cox model regression (OS bivariate analysis) showed the following factors associated with a worse prognosis for BL/L: population ageing, black race, disease in stage IV by Ann Arbor staging, no chemotherapy and no surgery on the primary site. No radiotherapy was associated with a good prognosis. Similar results were obtained with the multivariate analysis that additionally showed males associated with a worse OS prognosis when compared with females. In addition, the multivariate analysis showed that two primary sites (respiratory and cardiac systems and those parts related to the central nervous system: CNS) behaved as a worse prognosis for BL/L. All values of OS outcome (bivariate and multivariate analyses) are shown in Table 2.

### 3.3. Assessment of the Development Risk of Second Primary Cancers (SPCs)

**Figure 2:**
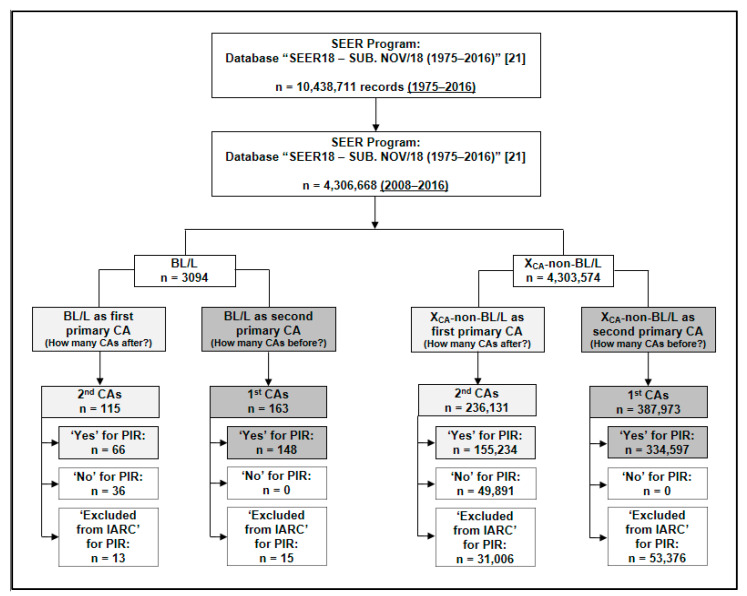
Data collection flowchart for the risk analysis of primary cancers before and after BL/L with BL/L behaving as an SPC and any cancer other than BL/L behaving as an SPC, respectively. Abbreviations: BL/L, Burkitt lymphoma/leukemia; CA, cancer; IARC, International Agency for Research on Cancer; PIR, primary cancer by international rules; SEER, Surveillance, Epidemiology and End Results; SPC, second primary cancer; X_CA_-non-BL/L, any cancer other than Burkitt lymphoma/leukemia.

Figure 2 shows the risk factor analysis flowchart. In the bivariate analysis, the first primary cancers that occurred before BL/L and behaved as risk factors for the BL/L occurrence as an SPC were anus, anal canal and anorectum tumors (RR, 4.2; 95% CI, 1.4–12.9; *p* = 0.017); Hodgkin lymphomas (HLs) (nodal) (RR, 9.9; 95% CI, 5.4–18.0; *p* < 0.001); Kaposi sarcomas (RR, 44.8; 95% CI, 23.6–85.0; *p* < 0.001); liver cancers (RR, 4.4; 95% CI, 1.7–11.5; *p* = 0.004) and trachea, mediastinum and other respiratory tumors (RR, 20.6; 95% CI, 2.9–146.2; *p* = 0.021). The median time until diagnosis of BL/L as an SPC was of five years (IQR, 2–9; range, 0–28).

BL/L behaved as a risk factor for the occurrence of the following SPCs: acute myeloid leukemia (AML) (RR, 9.9; 95% CI, 5.1–18.9; *p* < 0.001); anus, anal canal and anorectum tumors (RR, 6.4; 95% CI, 1.6–25.1; *p* = 0.017); HLs (extranodal) (RR, 168.0; 95% CI, 22.4–1259.4; *p* < 0.001) and Kaposi sarcomas (RR, 76.5; 95% CI, 29.1–201.0; *p* < 0.001). On the other hand, there was a decrease in the incidence of lung and bronchus cancer as an SPC (RR, 0.3; 95% CI, 0.1–0.8; *p* = 0.005). The median time until the presentation of other SPCs was of two years (IQR, 1–4; range, 0–8).

Lastly, the cancers that presented a *p*-value up to 0.25 in the bivariate analysis (see Supplementary Materials, Data S5) were tested for the risk cancers and were included in the Poisson regression model (see final model in Table 3).

## 4. Discussion

To our knowledge, this is the first population-based study that selected BL/L patients diagnosed with the most up-to-date WHOc [1,4,5], evaluated the prognostic factors associated with the OS and assessed what primary cancers were risk factors for the occurrence of BL/L as an SPC as well as if BL/L was a risk factor for the development of other SPCs. We found that BL/L patients had a mean OS of 65 months and a few factors as older age, advanced staging, no chemotherapy and radiotherapy were associated with a worse prognosis. Furthermore, we found that the main hematological cancers, Kaposi sarcomas and gastrointestinal tract (GIT) tumors were cancers with the risk of occurring before (BL/L as an SPC) or after (any cancer other than BL/L as an SPC) BL/L. Other population-based studies also using the SEER Program reported the occurrence of further malignancies after a previous cancer including prostate [28], breast [29] and chronic myeloid leukemia [30].

The international classification of BL was revised in 2008 and 2016. The cases initially classified as an atypical morphological variant of BL (‘Burkitt-like lymphoma’) in WHOc 2001 [3] were reallocated to the category DLBCL/BL in WHOc 2008 [4] and subsequently reclassified in WHOc 2016 as ‘high-grade B-cell lymphoma’. The latter may contain *MYC* and *BCL2* and/or *BCL6* rearrangements (“double-hit” or “triple-hit” lymphomas) and they usually have an aggressive clinical course and a worse prognosis than BL [1,5]. In our study, we restricted the date of the patient’s diagnosis (2008–2016) to prevent a misclassification of BL cases.

### 4.1. Prognostic Factors and Overall Survival (OS)

Our results showed that a few prognostic factors negatively impacted on the OS of BL/L patients. Aging was one of them. In the past decades, the significant increase in somatic mutations in animal and human cells damaged the genetic material of patients, resulting in a greater mortality from cancers at older ages including BL/L [31]. Studies showed that BL/L patients diagnosed <19 years old have a better prognosis (OS 5 years, 87%) than patients >60 years old or with an advanced tumor (OS 5 years, around 33%) [16,32].

Blacks were also associated with a worse prognosis for OS leading to a greater risk of dying from BL/L, which may be explained due to socioeconomic features. Wang et al. (2008) studied elderly patients with NHL and highlighted that African-Americans presented a lower educational level, income and socioeconomic status than Caucasian patients. Consequently, the proportion of patients on chemotherapy was lower among blacks and a poorer socioeconomic status was significantly associated with a higher risk of mortality [33]. However, a few studies have shown that a poorer prognosis regardless of the socioeconomic status would involve a host susceptibility factor, as observed in eBL in Africa [15].

Almost half of the patients in our study presented stage IV BL. Liu et al. (2019) showed the difficulty in finding BL cases at earlier stages (I and II) [34]. In our study, almost 60% of BL patients had a nodal presentation. A Chinese study reported sBL patients with a greater disease involvement at extranodal sites [35] while a Spanish study found lymph nodes to be the most frequently involved areas [36]. Similar epidemiological studies demonstrated that a nodal presentation in BL is commonplace in the American population [15,34]. These differences may have occurred due to the influence of the patient’s race, ethnicity, economic conditions and origin [37].

The bivariate analysis of the time-dependent covariate Cox regression showed the involvement of certain primary sites at diagnosis by BL/L as being statistically associated with a worse prognosis. The GIT was the second most frequently involved extranodal site as a primary diagnostic site while skin, breasts, the CNS and glands had less than 5% involvement each. sBL has an important abdominal involvement (affecting up to 5% of GIT NHL cases in the North American population) followed by the head and neck, nose, oropharynx and tonsils [7,38].

Patients not treated with chemotherapy or those who underwent radiotherapy had higher risks of death. Chemotherapy is the standard BL treatment [32]. Prophylactic CNS irradiation appears effective in acute lymphocytic leukemia and BL but is associated with a high toxicity, causing secondary malignant neoplasms and neuropsychological sequelae [39]. Surgery is not usually performed for this disease except when disease complications (e.g., intestinal obstruction) occur [32].

### 4.2. Risk of Second Primary Cancers (SPCs)

NHL comprises of a heterogeneous group of hematological diseases varying according to their biological characteristics. Each NHL subtype may have a different treatment, prognosis, clinical response and survival. Consequently, the risk for developing other cancers may also differ between these subtypes [1,17,40]. In both the bivariate and Poisson regression analyses, we found that anus, anal canal and anorectum tumors, HLs (nodal), Kaposi sarcomas, liver cancers and trachea, mediastinum and other respiratory cancers behaved as risk factors for the occurrence of BL/L as an SPC. One hypothesis is that these cancers involve sites that usually are affected as nodal or extranodal primary sites of BL [7]. On the other hand, BL/L behaved as a risk factor for the development of other SPCs such as AMLs, HLs (extranodal) and Kaposi sarcomas in the Poisson regression analysis. In addition, anus, anal canal and anorectum tumors presented a significant association with BL/L in the bivariate analysis.

There was an overall SPC risk 25% higher in patients who had a first NHL compared with the reference population. However, confounding factors such as age, sex, staging, follow-up time, number of chemotherapy agents and radiotherapy sessions might influence this outcome [17]. In addition, hormonal status and environmental and genetic factors may modify the risk of developing a second cancer [41] and should be considered during data analyses. AML occurrence as an SPC may be a late complication of cytotoxic therapy called ‘therapy-related myeloid neoplasm’ (t-MN). Alkylating agents (e.g., cyclophosphamide) and topoisomerase II inhibitors (e.g., etoposide) are chemotherapy agents that cause t-MN [19]. Kaposi sarcomas and BL/L have a high rate of incidence in HIV/AIDS individuals. Despite the change in pattern of secondary cancers after a Kaposi sarcoma in the era of antiretroviral therapy, NHL remains one of the most common [42,43].

We found that lung and bronchus cancers could decrease the occurrence of BL/L as an SPC as well as BL/L as a first primary cancer in the patient’s life decreased the incidence of these tumors and breast cancer as SPCs. These results should be interpreted with caution especially because our analysis did not consider the confounding factors. However, a recent study that investigated SPCs in breast cancer showed that the standardized incidence ratios in patients with chemotherapy were higher in all malignancies except lymphoma, myeloma and chronic lymphocytic leukemia [44].

### 4.3. Limitations

Our study has a few limitations. First, we cannot be absolutely sure that all BL/L patients in our study were diagnosed based on WHOc 2008 (or a later version) because this information was not available. With the date cutoff applied in the sample selection (2008–2016), we could infer that the most recent version of the WHOc may have been used. Second, the search date cutoff restricted the risk factor analysis to cancers that occurred after BL/L (that is, any cancer other than BL/L as an SPC). Third, the radiotherapy and chemotherapy variables were described according to the SEER*Stat as ‘No/Unknown’. The lack of descriptive information on these variables hampered further analyses. Fourth, information regarding aspects of treatment (e.g., drug name, radiation site), disease (e.g., relapse) and comorbidities (e.g., HIV) was not available; thus, other analyses including the prognosis of patients according to the type of treatment, evaluation of malignancies in patients with HIV (e.g., Kaposi sarcoma) or the distinction between the epidemiological clinical variants of BL could not be performed. Lastly, although our risk factor analysis presented several characteristics of a case-control study, factor and patient characteristics were not paired.

## 5. Conclusions

In conclusion, population ageing, black race, advanced staging, no chemotherapy, radiotherapy and no surgery were associated with a worse prognosis in BL/L, significantly decreasing the OS. First primary cancers that behaved as risk factors for the development of BL/L as an SPC were aleukemic, subleukemic and NOS tumors; anus, anal canal and anorectum tumors; HLs (nodal); Kaposi sarcomas; liver cancers and trachea, mediastinum and other respiratory organs cancers. On the other hand, lung and bronchus cancers decreased the incidence. At the same time, BL/L acted as a risk factor for the occurrence of other SPCs such as AML, HL (extranodal) and Kaposi sarcomas. On the other hand, a decreased incidence was observed for breast cancer and lung/bronchus tumors as SPCs. These results may assist the development of diagnostic and clinical recommendations in this field and guide the conduction of further studies on risk factors for hematological malignancies.

## Figures and Tables

**Table 1:** Sociodemographic, clinical and treatment characteristics of patients with BL/L.

Characteristics	*n* (%) (Total: *n* = 3094)
**Sociodemographic characteristics**	
**Age range (years old)**	
00–19	702 (22.7)
20–39	633 (20.5)
40–59	887 (28.7)
≥60	872 (28.2)
**Sex**	
Male	2255 (72.9)
Female	839 (27.1)
**Race**	
White	2458 (79.4)
Black	326 (10.5)
Other (American Indian/Alaska Native, Asian/Pacific Islander)	284 (9.2)
Unknown	26 (0.8)
**Origin**	
Non-Spanish-Hispanic-Latino	2451 (79.2)
Spanish-Hispanic-Latino	643 (20.8)
**Clinical characteristics**	
**Primary site ^1^**	
Lymph nodes	1798 (58.1)
Hematopoietic and reticuloendothelial systems	467 (15.1)
GIT and attachments	433 (14.0)
Lip, oral cavity and pharynx	112 (3.6)
Skin, bones, joints, articular cartilage and other tissues/sites	98 (3.2)
Breast and female/male genital organs	43 (1.4)
Eye, adnexa, brain and other parts of the CNS	36 (1.2)
Respiratory system and cardiac system and attachments	32 (1.0)
Glands (thyroid and adrenal)	31 (1.0)
Urinary system	15 (0.5)
Unknown primary site and other and ill-defined sites	29 (0.9)
**Ann Arbor staging ^2^**	
Stage I	448 (14.5)
Stage II	373 (12.1)
Stage III	270 (8.7)
Stage IV	1248 (40.3)
Others (Blank, Unknown, N/A)	755 (24.4)
**Treatment characteristics**	
**Chemotherapy**	
No/Unknown	389 (12.6)
Yes	2705 (87.4)
**Radiotherapy ^1^**	
No/Unknown	2912 (94.1)
Yes	182 (5.9)
**Surgery of primary site ^1^**	
No	1973 (63.8)
Yes (tumor destruction, resection, other types of surgery)	614 (19.8)
Unknown	507 (16.4)

Abbreviations: BL/L, Burkitt lymphoma/leukemia; CNS, central nervous system; GIT, gastrointestinal tract; N/A, not applicable. Notes: ^1^ Variable regrouped according to the similarities and number of events in each one of the groups (Supplementary Materials, Data S2). ^2^ Cases diagnosed until the year 2015. The background color was used to highlight the main group characteristics, while the bold highlighted the titles and subtitles.

**Table 2:** Bivariate and multivariate analyses by a time-dependent covariate Cox regression model.

Characteristics	Overall Survival						
	Bivariate Analysis			Multivariate Analysis			Deaths (%)
	HR	95% CI	*p*-Value *	HR	95% CI	*p*-Value *	
**Sociodemographic characteristics**							
**Age range (years old)**							
00–19	1.0	-	-	1.0	-	-	7.0
20–39	3.9	2.9–5.4	**<0.001**	3.5	2.5–4.8	**<0.001**	26.2
40–59	7.7	5.6–10.4	**<0.001**	6.1	4.5–8.4	**<0.001**	45.2
≥60	14.0	10.2–19.3	**<0.001**	10.5	7.5–14.5	**<0.001**	64.9
**Sex**							
Male	1.0	-	-	1.0	-	-	37.1
Female	1.1	0.9–1.2	0.390	0.9	0.8–1.0	**0.028**	40.9
**Race**							
White	1.0	-	-	1.0	-	-	37.4
Black	1.4	1.1–1.6	**0.001**	1.6	1.3–1.9	**<0.001**	45.8
Other ^1^	1.1	0.9–1.3	0.463	1.1	0.9–1.3	0.508	38.9
Unknown	0.2	0.1–0.8	**0.025**	0.2	0.1–0.8	**0.024**	7.7
**Origin**							
Spanish-Hispanic-Latino	1.0	-	-	1.0	-	-	31.1
Non-Spanish-Hispanic-Latino	1.2	1.0–1.4	**0.014**	1.0	0.8–1.1	0.592	40.0
**Clinical characteristics**							
**Histology/Behavior (ICD-O-3)**							
9687/3: Burkitt lymphoma	1.0	-	-	1.0	-	-	38.0
9826/3: Burkitt cell leukemia	1.1	1.0–1.4	0.110	0.8	0.5–1.3	0.346	39.1
**Primary site ^2^**							
Sites in lip, oral cavity and pharynx	1.0	-	-	1.0	-	-	17.0
GIT and attachments	2.4	1.5–3.8	**<0.001**	1.5	0.9–2.4	0.102	35.6
Respiratory system and cardiac system (with attachments)	3.1	1.6–6.2	**0.001**	2.1	1.1–4.2	**0.028**	46.9
Skin, bones, joints, articular cartilage and other tissues/sites	2.7	1.6–4.7	**<0.001**	1.6	0.9–2.7	0.096	42.9
Hematopoietic and reticuloendothelial systems	3.0	1.9–4.8	**<0.001**	0.8	0.2–3.1	0.699	41.3
Breast and female/male genital organs	2.5	1.3–4.7	**0.005**	1.5	0.8–2.8	0.224	44.2
Urinary system	2.1	0.8–5.2	0.123	1.1	0.4–2.7	0.899	40.0
Eye, adnexa, brain and other parts of the CNS	4.7	2.5–8.7	**<0.001**	2.2	1.2–4.2	**0.015**	58.3
Glands (thyroid and adrenal)	1.1	0.5–2.6	0.789	0.8	0.4–1.9	0.642	25.8
Lymph nodes	2.4	1.6–3.9	**<0.001**	1.4	0.9–2.3	0.127	38.4
Unknown primary site and other and ill-defined sites	3.2	1.6–6.5	**0.001**	0.5	0.1–2.3	0.382	44.8
**Ann Arbor staging ^3^**							
Stage I	1.0	-	-	1.0	-	-	29.8
Stage II	0.9	0.7–1.2	0.664	1.1	0.8–1.4	0.564	27.3
Stage III	1.1	0.8–1.4	0.592	1.2	0.9–1.6	0.140	31.5
Stage IV	1.9	1.5–2.3	**<0.001**	1.8	1.5–2.2	**<0.001**	48.0
Others (Blank, Unknown, N/A)	1.7	1.4–2.1	**<0.001**	1.5	1.1–2.0	**0.004**	34.6
**Treatment characteristics**							
**Chemotherapy**							
Yes	1.0	-	-	1.0	-	-	32.5
No/Unknown	4.9	4.3–5.7	**<0.001**	4.0	3.5–4.6	**<0.001**	78.4
**Radiotherapy ^2^**							
Yes	1.0	-	-	1.0	-	-	58.2
No/Unknown	0.7	0.6–0.8	**<0.001**	0.7	0.6–0.9	**0.001**	36.9
**Surgery of primary site ^2^**							
Yes (tumor destruction, resection, other types of surgery)	1.0	-	-	1.0	-	-	27.2
No	1.6	1.3–1.9	**<0.001**	1.4	1.1–1.6	**0.001**	40.7
Unknown	1.8	1.5–2.2	**<0.001**	3.5	1.0–12.8	0.058	41.8

Abbreviations: CI, confidence interval; CNS, central nervous system; GIT, gastrointestinal tract; ICD-O-3, International Classification of Diseases for Oncology, 3rd edition; HR, hazard ratio; N/A, not applicable. Notes: * *p*-value from the enter statistic test. ^1^ American Indian/AK Native, Asian/Pacific Islander. ^2^ Variable regrouped according to the similarities and number of events in each one of the groups (see Supplementary Materials, Data S2). ^3^ Cases diagnosed until 2015. The background color was used to highlight the main group characteristics, while the bold highlighted the titles, subtitles and significant *p*-values.

**Table 3:** Poisson regression final model of interrelated cancer risk on BL/L.

Cancers	First Primary Cancer				Second Primary Cancer			
	BL/L-2nd (%)	X_CA_-Non-BL/L-2nd (%)	RR (95% CI)	*p*-Value *	BL/L-1st (%)	X_CA_-Non-BL/L-1st (%)	RR (95% CI)	*p*-Value *
Aleukemic, subleukemic and NOS	1 (0.7)	184 (0.1)	9.5 (1.3–68.3)	**0.026**	-	-	-	-
AML	-	-	-	-	8 (12.1)	1909 (1.2)	4.6 (2.1–10.4)	**<0.001**
Anus, anal canal and anorectum	3 (2.0)	1615 (0.5)	3.2 (1.0–10.4)	**0.048**	2 (3.0)	736 (0.5)	3.0 (0.7–12.8)	0.134
Breast	14 (9.5)	43,177 (12.9)	0.6 (0.3–1.0)	0.059	1 (1.5)	9899 (6.4)	0.1 (0.0–0.8)	**0.032**
HL—extranodal	-	-	-	-	1 (1.5)	14 (0.01)	74.3 (10.0–549.8)	**<0.001**
HL—nodal	10 (6.8)	2288 (0.7)	7.6 (3.9–15.0)	**<0.001**	-	-	-	-
Kaposi sarcoma	9 (6.1)	454 (0.1)	34.0 (16.8–68.9)	**<0.001**	4 (6.1)	123 (0.1)	35.1 (12.1–101.4)	**<0.001**
Liver	4 (2.7)	2079 (0.6)	3.4 (1.2–9.3)	**0.020**	-	-	-	-
Lung and bronchus	4 (2.7)	20,642 (6.2)	0.3 (0.1–0.9)	**0.037**	3 (4.5)	26,604 (17.1)	0.1 (0.0–0.4)	**0.001**
Melanoma of the skin	4 (2.7)	15,905 (4.8)	0.4 (0.2–1.2)	0.113	-	-	-	-
Trachea, mediastinum and other respiratory organs	1 (0.7)	110 (0.03)	15.8 (2.2–113.9)	**0.006**	-	-	-	-

Abbreviations: AML, acute myeloid leukemia; BL/L, Burkitt lymphoma/leukemia; CI, confidence interval; HL, Hodgkin lymphoma; NOS, not otherwise specified; RR, relative risk; X_CA_-non-BL/L, any cancer other than BL/L. Notes: * *p*-value from the Wald statistic test. The bold was used to highlight the significant *p*-values.

## Data Availability

The data used in this study are available to the public in the references listed [21,22,23,24].

## Supplementary Materials

The following are available online at https://www.mdpi.com/article/10.3390/diseases9020043/s1, containing the items: Data S1. Definition of the variables (with Table S1); Data S2. Variables with their groups regrouped (with Data S2.1, Data S2.2 and Data S2.3); Data S3. Population characteristics of BL/L patients (with Figures S1 and S2); Data S4. Kaplan–Meier results (with Table S2); and Data S5. Bivariate analysis of risk factors of second primary cancer on BL/L (with Table S3).
