# Supplementary material for: Prognostic Factors, Survival Analyses and the Risk of Second Primary Cancer: A Population-Based Study on Burkitt Lymphoma/Leukemia
Source: Diseases. 2021 Jun 15;9(2):43. doi: 10.3390/diseases9020043 (PMC8293230; doi:10.3390/diseases9020043)
Supplement: Supplementary file 1 [file diseases-09-00043-s001.zip › diseases-1241807-supplementary.pdf]

SUPPLEMENTARY MATERIAL

Article title: *Prognostic factors, survival analyses and risk of second primary cancer: a population-based study on Burkitt lymphoma/leukemia*

Journal: *Diseases*

Summary

Data S1. Definition of the variables 2
Table S1. Definition of the variables from SEER Program 2
Data S2. Variables with their groups regrouped 3
Data S2.1. Primary site 3
Data S2.2. Surgery of primary site 5
Data S2.3. Radiotherapy 6
Data S3. Population characteristics of BL/L patients 7
Figure S1. Histogram of BL/L cases distribution according to age at diagnosis. 7
Figure S2. Number of alive and dead patients per year of diagnosis of BL/L population cohort. 7
Data S4. Kaplan-Meier results 8
Table S2. Results of the mean estimated overall survival according to sociodemographic, clinical and treatment characteristics of BL/L patients 8
Data S5. Bivariate analysis of risk factors of second primary cancer on BL/L 9
Table S3. Results from bivariate analysis of risk factors for second primary cancers 9

Data S1. Definition of the variables

Table S1. Definition of the variables from SEER Program

Variable	Definition
Age at diagnosis	It is the age of the patient at diagnosis for this cancer
Ann Arbor staging	It is a staging system widely used for anatomic staging of lymphoma, both HL and NHL. It is used for describe the extent of the disease
Chemotherapy	It informs if the chemotherapy treatment in the patient was performed or not
Histology/behavior	It reflects both the histology and the behavior of the tumor, according to ICD-O-3. The histologic type describes the microscopic composition of cells and/or tissue for a specific primary. The behavior is the fifth digit of the morphology code after the slash (/), being that: benign (/0), borderline (/1), in situ (/2) and malignant (/3)
Origin	It identifies the Spanish-Hispanic-Latino Ethnicity
Primary site	It identifies the site in which the primary tumor originated, according to topography codes from ICD-O-3
Primary cancer by international rules	It was created using IARC multiple primary rules and it did not include benign tumors or non-bladder in situ tumors in algorithm
Race	It is based on the race variable and the American Indian/Native American Indian Health Service link variable. This variable is independent of Hispanic ethnicity
Radiotherapy	It informs if the radiotherapy treatment (radiation) in the patient was performed or not
Record number	It sequentially numbers a person’s tumors. The ordered values are based on date of diagnosis and then sequence number
Sex	It identifies the sex of the patient at diagnosis
Site	It is based on Primary Site and ICD-O-3 Histology in order to make analyses of site/histology groups easier
Surgery of primary site	It describes a surgical procedure that removes and/or destroys tissue of the primary site performed as part of the initial work-up or first course of therapy
Survival	It was created using complete dates (including days), and it reflects the survival time in months
Vital status	It informs you if the patient is alive or if death occurred. This happens inside of follow-up cut-off date used in the study
Year of diagnosis	It reflects the year that the tumor was first diagnosed by a recognized medical practitioner, whether clinically or microscopically confirmed

HL, Hodgkin lymphoma; IARC, International Agency for Research on Cancer; ICD-10, International Classification of Diseases (10th version); ICD-O-3, International Classification of Diseases for Oncology – 3rd edition; NHL, non-Hodgkin lymphoma.

Data S2. Variables with their groups regrouped

The numbers in parentheses in item "Regrouped groups that were used in this study" correspond to the original groups of item "Variable groups according with the extraction from SEER*Stat software" inserted in the rearrangement performed.

Data S2.1. Primary site

Variable groups according with the extraction from SEER*Stat software:

- C80.9-Unknown primary site (0)
- C01.9-Base of tongue, NOS (1)
- C03.0-Upper gum (2)
- C03.9-Gum, NOS (3)
- C05.1-Soft palate, NOS (4)
- C06.0-Cheek mucosa (5)
- C06.2-Retromolar area (6)
- C06.9-Mouth, NOS (7)
- C07.9-Parotid gland (8)
- C08.0-Submandibular gland (9)
- C09.0-Tonsillar fossa (10)
- C09.9-Tonsil, NOS (11)
- C10.8-Overlapping lesion of oropharynx (12)
- C10.9-Oropharynx, NOS (13)
- C11.1-Posterior wall of nasopharynx (14)
- C11.3-Anterior wall of nasopharynx (15)
- C11.9-Nasopharynx, NOS (16)
- C13.9-Hypopharynx, NOS (17)
- C14.0-Pharynx, NOS (18)
- C14.2-Waldeyers ring (19)
- C14.8-Overlapping lesion of lip, oral cavity & pharynx (20)
- C15.5-Lower third of esophagus (21)
- C16.0-Cardia, NOS (22)
- C16.1-Fundus of stomach (23)
- C16.2-Body of stomach (24)
- C16.3-Gastric antrum (25)
- C16.4-Pylorus (26)
- C16.5-Lesser curvature of stomach NOS (27)
- C16.6-Greater curvature of stomach NOS (28)
- C16.8-Overlapping lesion of stomach (29)
- C16.9-Stomach, NOS (30)
- C17.0-Duodenum (31)
- C17.1-Jejunum (32)
- C17.2-Ileum (33)
- C17.8-Overlapping lesion of small intestine (34)
- C17.9-Small intestine, NOS (35)
- C18.0-Cecum (36)
- C18.1-Appendix (37)
- C18.2-Ascending colon (38)
- C18.3-Hepatic flexure of colon (39)
- C18.4-Transverse colon (40)
- C18.5-Splenic flexure of colon (41)
- C18.6-Descending colon (42)
- C18.7-Sigmoid colon (43)
- C18.8-Overlapping lesion of colon (44)
- C18.9-Colon, NOS (45)
- C19.9-Rectosigmoid junction (46)
- C20.9-Rectum, NOS (47)
- C21.0-Anus, NOS (48)
- C22.0-Liver (49)
- C23.9-Gallbladder (50)

- C25.0-Head of pancreas (51)
- C25.1-Body of pancreas (52)
- C25.9-Pancreas, NOS (53)
- C26.0-Intestinal tract, NOS (54)
- C26.8-Overlapping lesion of digestive system (55)
- C26.9-Gastrointestinal tract, NOS (56)
- C30.0-Nasal cavity (57)
- C31.0-Maxillary sinus (58)
- C31.1-Ethmoid sinus (59)
- C31.3-Sphenoid sinus (60)
- C31.8-Overlapping lesion of accessory sinuses (61)
- C31.9-Accessory sinus, NOS (62)
- C34.1-Upper lobe, lung (63)
- C34.2-Middle lobe, lung (64)
- C34.9-Lung, NOS (65)
- C38.0-Heart (66)
- C38.4-Pleura, NOS (67)
- C40.0-Long bones: upper limb, scapula, and associated joints (68)
- C40.2-Long bones of lower limb and associated joints (69)
- C41.0-Bones of skull and face and associated joints (70)
- C41.1-Mandible (71)
- C41.2-Vertebral column (72)
- C41.3-Rib, sternum, clavicle and associated joints (73)
- C41.4-Pelvic bones, sacrum, coccyx and associated joints (74)
- C41.9-Bone, NOS (75)
- C42.1-Bone marrow (76)
- C42.2-Spleen (77)
- C44.3-Skin other/unspec parts of face (78)
- C44.4-Skin of scalp and neck (79)
- C44.6-Skin of upper limb and shoulder (80)
- C44.7-Skin of lower limb and hip (81)
- C48.0-Retroperitoneum (82)
- C48.1-Specified parts of peritoneum (83)
- C48.2-Peritoneum, NOS (84)
- C48.8-Overlapping lesion of retropeiron (85)
- C49.0-Conn, subcutaneous, other soft tis: head, face, neck (86)
- C49.1-Coon, subcutaneous, other soft tis: upr limb, shoulder (87)
- C49.2-Conn, subcutaneous, other soft tis: lower limb, hip (88)
- C49.3-Conn, subcutaneous, other soft tis: thorax (89)
- C49.4-Conn, subcutaneous, other soft tis: abdomen (90)
- C49.5-Conn, subcutaneous, other soft tis: pelvis (91)
- C49.6-Conn, subcutaneous, other soft tis: trunk, NOS (92)
- C49.8-Overlap conn, subcutaneous, and other soft tissues (93)
- C49.9-Conn, subcutaneous and other soft tissues, NOS (94)
- C50.1-Central portion of breast (95)
- C50.2-Upper-inner quadrant of breast (96)
- C50.4-Upper-outer quadrant of breast (97)
- C50.8-Overlapping lesion of breast (98)
- C50.9-Breast, NOS (99)
- C51.8-Overlapping lesion of vulva (100)
- C53.9-Cervix uteri (101)
- C55.9-Uterus, NOS (102)
- C56.9-Ovary (103)
- C57.7-Other specified parts of female genital organs (104)
- C57.8-Overlapping lesion of female genital organs (105)
- C62.1-Descended testis (106)
- C62.9-Testis, NOS (107)
- C64.9-Kidney, NOS (108)
- C67.4-Posterior wall of bladder (109)
- C67.9-Bladder, NOS (110)

- C68.0-Urethra (111)
- C69.6-Orbit, NOS (112)
- C69.9-Eye, NOS (113)
- C71.1-Frontal lobe (114)
- C71.3-Parietal lobe (115)
- C71.4-Occipital lobe (116)
- C71.5-Ventricle, NOS (117)
- C71.6-Cerebellum, NOS (118)
- C71.7-Brain stem (119)
- C71.8-Overlapping lesion of brain (120)
- C71.9-Brain, NOS (121)
- C72.0-Spinal cord (122)
- C72.9-Nervous system, NOS (123)
- C73.9-Thyroid gland (124)
- C74.9-Adrenal gland, NOS (125)
- C76.0-Head, face or neck, NOS (126)
- C76.1-Thorax, NOS (127)
- C76.2-Abdomen, NOS (128)
- C77.0-Lymph nodes of head, face & neck (129)
- C77.1-Intrathoracic lymph nodes (130)
- C77.2-Intra-abdominal lymph nodes (131)
- C77.3-Lymph nodes of axila or arm (132)
- C77.4-Lymph nodes of inguinal region or leg (133)
- C77.5-Pelvic lymph nodes (134)
- C77.8-Lymph nodes of multiple regions (135)
- C77.9-Lymph node, NOS (136)

Regrouped groups that were used in this study:

- Lip, oral cavity and pharynx (1 to 20)
- Gastrointestinal tract and attachments (21 to 56)
- Respiratory system and cardiac system, and attachments (57 to 67)
- Skin, bones, joints, articular cartilage and other tissues/sites (68 to 75; 78 to 94)
- Hematopoietic and reticuloendothelial systems (76, 77)
- Breast and female/male genital organs (95 to 107)
- Urinary system (108 to 111)
- Eye, adnexa, brain and other parts of central nervous system (112 to 123)
- Glands (thyroid and adrenal) (124, 125)
- Lymph nodes (129 to 136)
- Unknown primary site and other and ill-defined sites (0; 126 to 128)

Data S2.2. Surgery of primary site

Variable groups according with the extraction from SEER*Stat software:

- 00 - None; no surgical procedure of primary site; diagnosed at autopsy only (0)
- 10-19 - Site-specific codes. Tumor destruction; no pathologic specimen or unknown whether there is a pathologic specimen (1)
- 20-80 - Site-specific codes. Resection; pathologic specimen (2)
- 90 - Surgery, NOS. Surg proced to the primary site was done, but no information type of surg procedure (3)
- 98 - All hematopoietic / reticuloendothelial / immunoproliferative / myeloproliferative disease sites and/or histology, WITH OR WITHOUT surgical treatment; all unknown and ill-defined disease sites, WITH OR WITHOUT surgical treatment; and unknown primaries, except death certificate only (4)
- 99 - Unknown if surgery performed (5)

Regrouped groups that were used in this study:

- No (0)
- Yes (tumor destruction, resection, other types of surgery) (1 to 3)
- Unknown (4, 5)

Data S2.3. Radiotherapy

Variable groups according with the extraction from SEER*Stat software:

- None/Unknown (0)
- Refused (1988+) (1)
- Recommended, unknown if administered (2)
- Radiation, NOS method or source not specified (3)
- Beam radiation (4)

Regrouped groups that were used in this study:

- None/Unknown (0 to 2)
- Yes (3, 4)

Data S3. Population characteristics of BL/L patients

Figure S1. Histogram of BL/L cases distribution according to age at diagnosis.

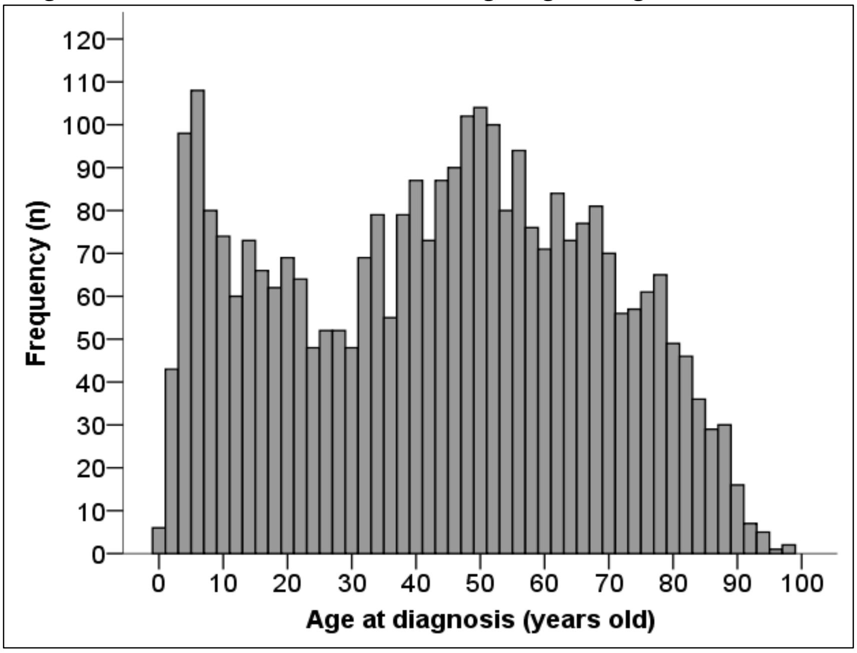

BL/L, Burkitt lymphoma/leukemia.

Figure S2. Number of alive and dead patients per year of diagnosis of BL/L population cohort.

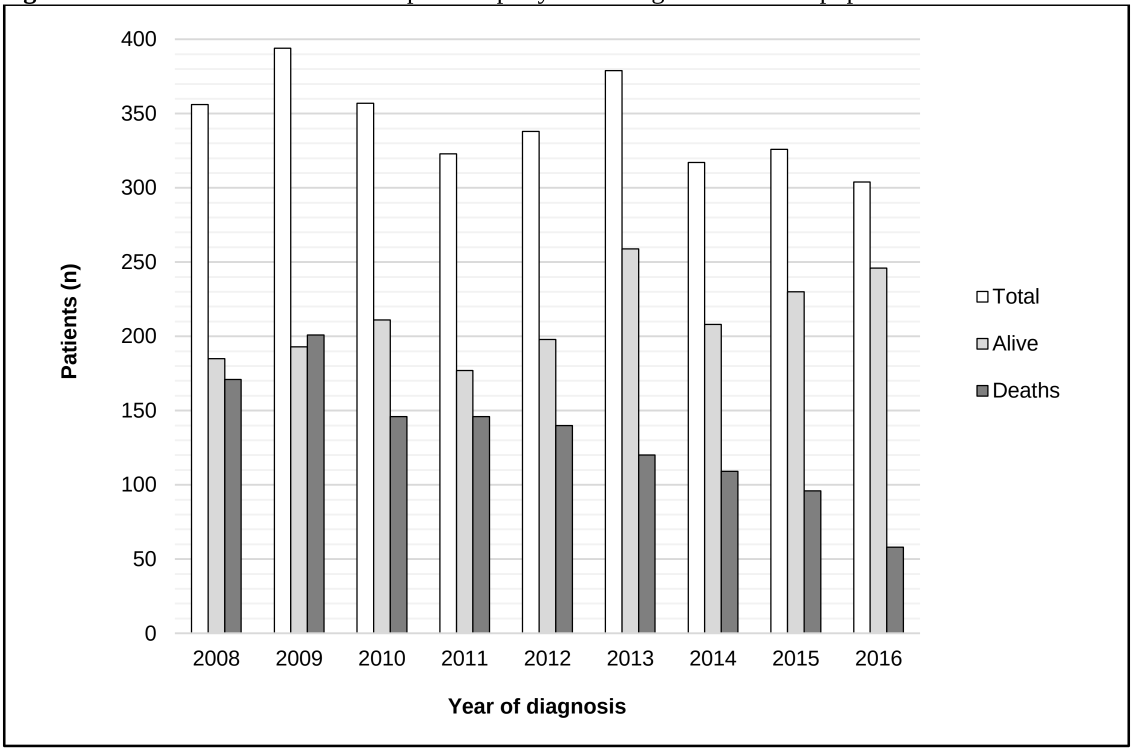

BL/L, Burkitt lymphoma/leukemia.

Data S4. Kaplan-Meier results

Table S2. Results of the mean estimated overall survival according to sociodemographic, clinical and treatment characteristics of BL/L patients

Independent variable	Overall survival (months) ('vital status' variable)					
	**M**	**SE**	**95% CI**	***p*-value (LR)**	***p*-value (BR)**	***p*-value (TW)**
General	65.4	0.9	63.6-67.3	-	-	-
**Sociodemographic characteristics**						
**Age range (years old)**						
00-19	98.9	1.1	96.8-101.1	**< 0.001**	**< 0.001**	**< 0.001**
20-39	78.1	1.9	74.3-81.8			
40-59	59.2	1.7	55.7-62.6			
≥ 60	37.6	1.7	34.3-40.8			
**Sex**						
Male	66.5	1.1	64.4-68.7	0.096	0.292	0.169
Female	62.5	1.8	59.0-66.1			
**Race**						
White	66.3	1.0	64.2-68.3	**0.001**	**0.001**	**0.001**
Black	57.8	2.9	52.1-63.5			
Other (American Indian/AK Native, Asian/Pacific Islander)	64.2	3.1	58.1-70.2			
Unknown	73.5	5.7	62.4-84.6			
**Origin**						
Spanish-Hispanic-Latino	71.7	2.0	67.7-75.7	**0.001**	**0.003**	**0.002**
Non-Spanish-Hispanic-Latino	63.9	1.0	61.9-66.0			
**Clinical characteristics**						
**Histology/Behavior (ICD-O-3)**						
9687/3: Burkitt lymphoma	66.0	1.0	64.0-67.9	0.333	0.920	0.701
9826/3: Burkitt cell leukemia	60.4	2.8	54.9-66.0			
**Primary site**[1]						
Sites in lip, oral cavity and pharynx	89.2	3.7	82.0-96.5	**< 0.001**	**< 0.001**	**< 0.001**
GIT and attachments	67.7	2.5	62.8-72.6			
Respiratory system and cardiac system (with attachments)	57.3	8.7	40.2-74.3			
Skin, bones, joints, articular cartilage and other tissues/sites	61.8	5.2	51.5-72.0			
Hematopoietic and reticuloendothelial systems	57.7	2.7	52.5-62.9			
Breast and female/male genital organs	57.6	7.7	42.5-72.7			
Urinary system	66.6	11.6	43.9-89.3			
Eye, adnexa, brain and other parts of CNS	39.7	8.8	22.5-57.0			
Glands (thyroid and adrenal)	78.8	7.7	63.7-93.9			
Lymph nodes	65.8	1.2	63.4-68.1			
Unknown primary site and other and ill-defined sites	55.5	8.8	38.3-72.7			
**Ann Arbor staging**[2]						
Stage I	76.9	2.2	72.7-81.2	**< 0.001**	**< 0.001**	**< 0.001**
Stage II	78.6	2.4	73.9-83.3			
Stage III	75.2	2.8	69.6-80.7			
Stage IV	57.2	1.5	54.4-60.1			
Others (Blank, Unknown, N/A)	58.9	2.4	54.2-63.6			
**Treatment characteristics**						
**Chemotherapy**						
No/Unknown	20.6	2.2	16.4-24.8	**< 0.001**	**< 0.001**	**< 0.001**
Yes	71.6	1.0	69.7-73.4			
**Radiotherapy**[1]						
No/Unknown	66.7	1.0	64.8-68.6	**< 0.001**	**0.004**	**< 0.001**
Yes	47.1	3.6	40.1-54.2			
**Surgery of primary site**[1]						
No	63.1	1.2	60.8-65.4	**< 0.001**	**< 0.001**	**< 0.001**
Yes (tumor destruction, resection, other types of surgery)	77.7	1.9	74.0-81.5			
Unknown	57.6	2.6	52.6-62.6			

ALL, acute lymphocytic leukemia; BL/L, Burkitt lymphoma/leukemia; BR, Breslow (Generalized Wilcoxon); CI, confidence interval; CNS, central nervous system; GIT, gastrointestinal tract; LR, Log-Rank (Mantel-Cox); ICD-O-3, International Classification of Diseases for Oncology – 3rd edition; M, meantime survival (months); NHL, non-Hodgkin lymphoma; SE, standard error; TW, Tarone-Ware.

[1]Variable regrouped according to the similarities and number of events in each one of groups (to see *Supplementary material – Data S2*).

[2]Cases diagnosed until the year 2015.

Data S5. Bivariate analysis of risk factors of second primary cancer on BL/L

Table S3. Results from bivariate analysis of risk factors for second primary cancers (continues)

First (antecedent) and second primary cancer (subsequent)	1st cancers – 'Yes' for PIR[1] (Antecedent cancers)				2nd cancers – 'Yes' for PIR[1] (Subsequent cancers)			
	Cases (n = 148)	***Controls*** (n = 334,597)			***Cases*** (n = 66)	***Controls*** (n = 155,234)		
	**BL/L-2nd – n (%)**	**X_{CA}-non-BL/L-2nd – n (%)**	**RR (95% CI)**	***p*-value**	**BL/L-1st – n (%)**	**X_{CA}-non-BL/L-1st – n (%)**	**RR (95% CI)**	***p*-value**
Acute myeloid leukemia	-	-	-	-	8 (12.1)	1,909 (1.2)	9.9 (5.1-18.9)	**< 0.001**
Aleukemic, subleukemic and NOS	1 (0.7)	184 (0.1)	12.3 (1.7-87.1)	0.072	-	-	-	-
Anus, anal canal and anorectum	3 (2.0)	1,615 (0.5)	4.2 (1.4-12.9)	**0.017**	2 (3.0)	736 (0.5)	6.4 (1.6-25.1)	**0.017**
Ascending colon	3 (2.0)	4,743 (1.4)	1.4 (0.5-4.4)	0.390	2 (3.0)	2,042 (1.3)	2.3 (0.6-9.0)	0.248
Brain	-	-	-	-	1 (1.5)	1,396 (0.9)	1.7 (0.2-11.8)	0.451
Breast	14 (9.5)	43,177 (12.9)	0.7 (0.5-1.2)	0.130	1 (1.5)	9,899 (6.4)	0.2 (0.0-1.7)	0.086
Cecum	2 (1.4)	5,285 (1.6)	0.9 (0.2-3.4)	0.457	2 (3.0)	2,199 (1.4)	2.1 (0.6-8.4)	0.278
Chronic myeloid leukemia	1 (0.7)	987 (0.3)	2.3 (0.3-16.2)	0.462	1 (1.5)	649 (0.4)	3.6 (0.5-25.4)	0.335
Corpus uteri	5 (3.4)	12,731 (3.8)	0.9 (0.4-2.1)	0.478	1 (1.5)	4,246 (2.7)	0.6 (0.1-3.9)	0.409
Cranial nerves other nervous system	1 (0.7)	279 (0.1)	8.1 (1.1-57.4)	0.142	-	-	-	-
Descending colon	1 (0.7)	1,556 (0.5)	1.5 (0.2-10.3)	0.410	-	-	-	-
Esophagus	-	-	-	-	1 (1.5)	1,999 (1.3)	1.2 (0.2-8.2)	0.351
Eye and orbit	1 (0.7)	786 (0.2)	2.9 (0.4-20.3)	0.398	-	-	-	-
Gallbladder	1 (0.7)	408 (0.1)	5.5 (0.8-39.2)	0.226	-	-	-	-
Hodgkin lymphoma – extranodal	-	-	-	-	1 (1.5)	14 (0.01)	168.0 (22.4-1,259.4)	**< 0.001**
Hodgkin lymphoma – nodal	10 (6.8)	2,288 (0.7)	9.9 (5.4-18.0)	**< 0.001**	1 (1.5)	467 (0.3)	5.0 (0.7-35.3)	0.249
Kaposi sarcoma	9 (6.1)	454 (0.1)	44.8 (23.6-85.0)	**< 0.001**	4 (6.1)	123 (0.1)	76.5 (29.1-201.0)	**< 0.001**
Kidney and renal pelvis	5 (3.4)	12,543 (3.7)	0.9 (0.4-2.1)	0.492	7 (10.6)	9,217 (5.9)	1.8 (0.9-3.6)	0.090
Large intestine, NOS	-	-	-	-	1 (1.5)	640 (0.4)	3.7 (0.5-25.7)	0.331
Larynx	1 (0.7)	5,216 (1.6)	0.4 (0.1-3.1)	0.296	-	-	-	-

Table S3. Results from bivariate analysis of risk factors for second primary cancers (conclusion)

First (antecedent) and second primary cancer (subsequent)	1st cancers – 'Yes' for PIR[1] (Antecedent cancers)				2nd cancers – 'Yes' for PIR[1] (Subsequent cancers)			
	Cases (n = 148) **BL/L-2nd – n (%)**	***Controls*** (n = 334,597) **X_{CA}-non-BL/L-2nd – n (%)**	**RR (95% CI)**	***p*-value**	***Cases*** (n = 66) **BL/L-1st – n (%)**	***Controls*** (n = 155,234) **X_{CA}-non-BL/L-1st – n (%)**	**RR (95% CI)**	***p*-value**
Liver	4 (2.7)	2,079 (0.6)	4.4 (1.7-11.5)	**0.004**	-	-	-	-
Lung and bronchus	4 (2.7)	20,642 (6.2)	0.4 (0.2-1.2)	0.057	3 (4.5)	26,604 (17.1)	0.3 (0.1-0.8)	**0.005**
Melanoma of the skin	4 (2.7)	15,905 (4.8)	0.6 (0.2-1.5)	0.164	2 (3.0)	6,112 (3.9)	0.8 (0.2-3.0)	0.475
Miscellaneous	4 (2.7)	6,810 (2.0)	1.3 (0.5-3.5)	0.388	5 (7.6)	6,075 (3.9)	1.9 (0.8-4.5)	0.112
Non-Hodgkin lymphoma – extranodal	2 (1.4)	5,029 (1.5)	0.9 (0.2-3.6)	0.426	-	-	-	-
Ovary	1 (0.7)	4,282 (1.3)	0.5 (0.1-3.7)	0.387	-	-	-	-
Pancreas	1 (0.7)	2,044 (0.6)	1.1 (0.2-7.8)	0.335	3 (4.5)	4,964 (3.2)	1.4 (0.5-4.3)	0.393
Prostate	41 (27.7)	73,445 (22.0)	1.3 (1.0-1.6)	0.056	5 (7.6)	15,346 (9.9)	0.8 (0.3-1.8)	0.336
Rectosigmoid juction	-	-	-	-	1 (1.5)	1,197 (0.8)	2.0 (0.3-13.8)	0.495
Rectum	6 (4.1)	8,051 (2.4)	1.7 (0.8-3.7)	0.149	-	-	-	-
Salivary gland	-	-	-	-	1 (1.5)	556 (0.4)	4.2 (0.6-29.6)	0.294
Sigmoid colon	1 (0.7)	8,150 (2.4)	0.3 (0.0-2.0)	0.131	3 (4.5)	2,581 (1.7)	2.7 (0.9-8.3)	0.089
Soft tissue including heart	3 (2.0)	2,271 (0.7)	3.0 (1.0-9.2)	0.067	-	-	-	-
Stomach	2 (1.4)	3,378 (1.0)	1.3 (0.3-5.3)	0.498	1 (1.5)	3,053 (2.0)	0.8 (0.1-5.4)	0.429
Thyroid	4 (2.7)	8,816 (2.6)	1.0 (0.4-2.7)	0.419	5 (7.6)	5,212 (3.4)	2.3 (1.0-5.2)	0.059
Tongue	1 (0.7)	3,353 (1.0)	0.7 (0.1-4.8)	0.494	1 (1.5)	1,432 (0.9)	1.6 (0.2-11.5)	0.444
Tonsil	1 (0.7)	2,119 (0.6)	1.1 (0.2-7.5)	0.325	-	-	-	-
Trachea, mediastinum and other respiratory organs	1 (0.7)	110 (0.03)	20.6 (2.9-146.2)	**0.021**	-	-	-	-
Urinary bladder	10 (6.8)	23,582 (7.0)	1.0 (0.5-1.7)	0.491	3 (4.5)	9,034 (5.8)	0.8 (0.3-2.4)	0.429

BL/L, Burkitt lymphoma/leukemia; NOS, not otherwise specified; PIR, primary cancer by international rules; RR, relative risk; X_{CA}-non-BL/L, any cancer other than BL/L.
[1]International rules from the *International Agency for Research on Cancer* (IARC).
